# Effectiveness of the 2012/13 Trivalent Live and Inactivated Influenza Vaccines in Children and Adolescents in Saxony-Anhalt, Germany: A Test-Negative Case-Control Study

**DOI:** 10.1371/journal.pone.0122910

**Published:** 2015-04-17

**Authors:** Carina Helmeke, Lutz Gräfe, Hanns-Martin Irmscher, Constanze Gottschalk, Ioannis Karagiannis, Hanna Oppermann

**Affiliations:** 1 European Programme for Intervention Epidemiology Training (EPIET), European Centre for Disease Prevention and Control (ECDC), Stockholm, Sweden; 2 State Agency for Consumer Protection Saxony-Anhalt, Department of Hygiene, Magdeburg, Germany; Centers for Disease Control and Prevention, UNITED STATES

## Abstract

A live attenuated influenza vaccine has been available in Germany since the influenza season 2012/13, which is approved for children aged 2-17 years. Using data from our laboratory-based surveillance system, we described the circulation of influenza and non-influenza respiratory viruses during the influenza season 2012/13 in Saxony-Anhalt. We estimated the effectiveness of live and inactivated trivalent influenza vaccines in preventing laboratory-confirmed cases among children and adolescents. From week 40/2012 to 19/2013, sentinel paediatricians systematically swabbed acute respiratory illness patients for testing of influenza and 5 non-influenza viruses by PCR. We compared influenza cases and influenza-negative controls. Among children aged 2-17 years, we calculated overall and vaccine type-specific effectiveness against laboratory-confirmed influenza, stratified by age group (2-6; 7-17 years). We used multivariable logistic regression to adjust estimates for age group, sex and month of illness. Out of 1,307 specimens, 647 (35%) were positive for influenza viruses and 189 (15%) for at least one of the tested non-influenza viruses. For vaccine effectiveness estimation, we included 834 patients (mean age 7.3 years, 53% males) in our analysis. Of 347 (42%) influenza-positive specimens, 61 (18%) were positive for A(H1N1)pdm09, 112 (32%) for A(H3N2) and 174 (50%) for influenza B virus. The adjusted overall vaccine effectiveness including both age groups was 38% (95% CI: 0.8-61%). The adjusted effectiveness for inactivated vaccines was 37% (95% CI: -35-70%) and for live vaccines 84% (95% CI: 45-95%). Effectiveness for the live vaccine was higher in 2-6 year-old children (90%, 95% CI: 20-99%) than in children aged 7-17 years (74%, 95% CI: -32-95%). Our study of the strong influenza season in 2012/13 suggests a high preventive effect of live attenuated influenza vaccine especially among young children, which could not be reached by inactivated vaccines. We recommend the use of live attenuated influenza vaccines in children unless there are contraindications.

## Introduction

The World Health Organization (WHO) recommends the antigen combination for the seasonal influenza vaccines in the northern hemisphere annually every February. In the 2012/13 season, the trivalent vaccines contained the following strains: an A/California/7/2009(H1N1)-like strain, an A/Victoria/361/2011(H3N2)-like strain and a B/Wisconsin/1/2010-like strain (Yamagata-line) [1]. Because of the continuous variation of influenza viruses, it is necessary to evaluate the effectiveness of influenza vaccines seasonally. Additionally to the trivalent inactivated influenza vaccines (TIV), a nasal, trivalent live attenuated influenza vaccine (LAIV) has been used in the U.S. under the name FluMist since 2003; this was later approved in Canada and the European Union [2,3]. Various randomized trials estimated a better efficacy of LAIV compared to TIV and a good toleration of LAIV in children [2–8].

In Germany, influenza vaccination is, as all other vaccinations, voluntary, but the Standing Committee on Vaccination (STIKO) recommends annual influenza vaccination for certain target groups [9]. Healthy children and adolescents do not belong to a target group, however if they want to be protected they need to be vaccinated. According to a study of Böhmer et al. [10], influenza vaccination coverage among people who did not belong to a STIKO-targeted group was 15% in 2010/11. Since the influenza season 2012/13, a LAIV (trade name Fluenz has been available in addition to TIV. Fluenz is approved for children aged 2 to 17 years. The STIKO recently adapted its recommendation for influenza vaccination: children and adolescents aged 2 to 17 years may be vaccinated with an inactivated vaccine or with a LAIV, unless there are contraindications. In children aged 2 to 6 years, LAIV should be used preferentially [11].

Since 2007, the German federal state of Saxony-Anhalt has an established virological surveillance system for monitoring of influenza and other respiratory viruses and to produce estimation of influenza vaccine effectiveness. Ten percent of the population of Saxony-Anhalt lives in the district of the capital Magdeburg (200 thousand of 2 million people). The virological data are regularly sent to the national influenza surveillance at the Robert Koch-Institute (RKI) and contribute to the Influenza Monitoring Vaccine Effectiveness (I-MOVE) network. I-MOVE measures influenza VE conducting multi-centre studies including data of all member states of their European network. To our knowledge, I-Move did not estimate influenza VE stratified by vaccine type (LAIV and TIV) for the season 2012/13 (E. Kissling personal communication).

The aims of our study were to describe the circulation of respiratory viruses and to determine the vaccine effectiveness (VE) of LAIV and non-adjuvant TIV against laboratory-confirmed influenza illness in children and adolescents during the season 2012/13 in Saxony-Anhalt. Here, we provide VE estimates based on data collected by our protocol used in Saxony-Anhalt exclusively (one-centre study).

## Materials and Methods

### Ethics statement

Paediatricians obtained informed verbal consent from the next of kin, caretakers, or guardians on behalf of the minors/children enrolled in the surveillance. Informed consent was documented with the signature of the paediatrician on the surveillance questionnaire.

Since patients would have been swabbed for routine diagnostic purposes as well, no additional risks were presented requiring written consent of the patient. Therefore, written consent of the patient was not obtained. This consent procedure was approved by the responsible ethics committee in Saxony-Anhalt.

### Data sources

#### Sampling and description of study population

We used data between calendar weeks (CW) 40 in 2012 to CW 19 in 2013. Participating paediatricians in primary care in Saxony-Anhalt systematically swabbed the throat or nasopharynx of patients presented with acute respiratory illness (ARI) according to predetermined selection criteria. ARI patients were swabbed when a confirmation that the disease was caused viral, was significant for further treatment. The number of patients who refused to be swabbed was not documented. We defined ARI patients as patients with an acute onset of medically-confirmed respiratory symptoms. A patient was classified as vaccinated if they had had at least one dose of seasonal influenza vaccine. The sentinel paediatricians completed a standardised questionnaire collecting demographic, clinical and specimen information at the time of swabbing by interviewing patients or their parents. Clinical information was supplemented by the paediatrician and derived from patient records and vaccination cards. A routine courier transported specimens and questionnaires to our laboratory (usually on the day of swabbing).

#### Virological investigation

Our laboratory analysed specimens by real-time polymerase chain reaction (PCR). We performed automated viral nucleic acid extraction using Invisorb Virus RNA HTS 96 Kit (STRATEC molecular, Germany) on CAS-1820 (Corbett Life Science). We performed reverse transcription PCR assays for the detection of influenza A and B viruses for each specimen [12,13]. In case of initial positive influenza virus A results, we performed further subtyping for A(H3N2) and A(H1N1)pdm09 using confirmatory reverse transcription PCR assays [14,15]. For each influenza-negative specimen, we performed adenovirus PCR [16], respiratory syncytial virus (RSV) reverse transcription PCR [17,18], human metapneumovirus (HMPV) [19,20], rhinovirus PCR [21]) and enterovirus reverse transcription PCR [22,23]. We adapted all PCR assays to our own laboratory conditions using Real Time Cycler Rotor-Gene 3000 or 6000 (Corbett Life Science) and automated liquid handling on CAS-1200 (Corbett Life Science). In addition, we inoculated all specimens on Madin-Darby Canine Kidney (MDCK) cells for influenza virus isolation. To compare the circulating virus strains with the components contained in the influenza vaccines, we sent selected isolates to the National Reference Center (NRC) for influenza in Berlin for further characterization.

### Analytical study

#### Study population

We included ARI patients aged 2 to 17 years in our analysis. We excluded data from patients under 2 and over 17 years in order to ensure the comparability of our data with the age group for whom LAIV is approved in Germany. Patients with respiratory symptoms but no acute onset of illness did not meet the ARI definition. We excluded data from patients from our analysis when 1) respiratory illness had no acute onset; 2) vaccination status was unknown (for LAIV and TIV vaccine effectiveness estimation also when the name of the vaccine was unknown); 3) documented vaccination was within 14 days before disease onset; 4) it was known that sampling was performed more than 8 days after disease onset. We defined the month of swabbing as month of illness if the date of onset of illness was missing.

#### Study design

We conducted a test-negative case-control study, comparing laboratory-confirmed influenza cases with influenza-negative controls in accordance to the ECDC Protocol [24–26]. We defined a case of influenza as a medically attended ARI patient whose swab tested positive for influenza virus A or B by PCR or virus isolation in our laboratory. Controls were classified as medically-attended ARI patients with swabs testing negative for influenza virus.

#### Vaccine effectiveness

We estimated VE as (1-odds ratio (OR)) x 100%. We calculated the effectiveness of any seasonal vaccination (all vaccines) and of LAIV and TIV in children aged 2 to 17 years and stratified by age groups (2–6, 7–17 years). We used multivariable logistic regression to adjust VE estimates for age group, sex and month of illness.

Different characteristics of cases and controls were analysed using chi-square test and, for continuous variables, using median test.

## Results

### Data sources

#### Sampling and description of study population

Fifteen paediatric practices with 17 paediatricians in 7 districts, representing 9.4% of the paediatricians in Saxony-Anhalt participated in our study. In 1280 (98%) of 1,307 samples, information was completed on age (mean: 6.3 years). Among patients with adequate information, 53% were male, 94% with acute onset of illness and 9.4% were vaccinated (Table 1).

**Table 1:** Data characteristics of patients swabbed during the study period from week 40/2012 to 19/2013, Virological Surveillance, Saxony-Anhalt.

	With information		Among them with information	
	n	%	n	%
**Total number of samples**	1,307	100		
**Male**	1,294	99	684	53
**Acute onset of illness**	1,261	96	1,182	94
**Vaccinated**	1,202	92	113	9.4

#### Virological investigation

The first specimens were tested influenza-positive in CW 48 in 2012. The percentage of influenza—positive specimens reached its peak at 5th until 9th CW 2013 (up to 56%) and decreased until CW 19. Out of 1,307 sentinel specimens 458 (35%) were positive for influenza viruses and 189 (15%) for at least one of the tested non-influenza viruses (Table 2). The percentage of RSV-positive specimens was 8%, of adenovirus-positive specimens 3.1%, of HMPV-positive specimens 1.3%, of rhinovirus-positive specimens 1.1% and of enterovirus-positive specimens 0.92%.

**Table 2:** Laboratory results from specimens of patients swabbed during the study period from week 40/2012 to 19/2013, Virological Surveillance, Saxony-Anhalt.

	n	%
**Number of specimens**	1307	100
**Total number positive**	647	50
**Influenza virus**	458	35
A(H1N1)pdm09	98	7.5
A(H3N2)	148	11
Influenza B	212	16
**RSV** *	105	8.0
hMPV*	17	1.3
**Adeno**	40	3.1
**Picornavirus**	27	2.1
Enterovirus	12	0.9
Rhinovirus	15	1.1

*RSV (respiratory syncytial virus), HMPV (human metapneumovirus)

Among 458 influenza virus-positive specimens, influenza B viruses dominated in 212 (46%) of the samples. Seasonal influenza A(H3N2) viruses and pandemic influenza A viruses were detected in 148 (32%) and in 98 (21%) respectively of influenza virus-positive specimens. From 53 selected influenza virus isolates characterized at the NRC, 24 (45%) tested positive for A/Victoria/361/2011(H3N2)-like, 19 (36%) for A/California/7/09-like, and 10 (19%) for B/Estonia/55669/2011-like (Yamagata-line) strains.

Out of 458 PCR-tested influenza virus-positive specimens, 306 (67%) were confirmed by culture: 78 (80%) of A(H1N1)pdm09, 142 (77%) of influenza virus B and 86 (58%) of A(H3N2). In few cases with PCR-negative and culture-positive results, repetition of PCR gained positive results too.

### Analytical study

#### Study population

Of 1,307 patients, 301 were aged younger than 2 years and 13 older than 17 years. For 4 patients, sampling was performed more than 8 days after disease onset. In 125 patient questionnaires, there was no evidence for acute symptom onset. In some patient questionnaires information was missing on vaccination status (n = 105), on age (n = 27) and on sex (n = 13). After excluding these data of 473 patients, 834 (64%) were included in our analytical study. Out of the 834 patients, 347 (42%) were laboratory-confirmed influenza cases. All influenza viruses were subtyped. Among 347 influenza viruses 174 (50%) were positive for influenza B viruses, 112 (32%) for A(H3N2) and 61 (18%) for A(H1N1)pdm09. Influenza cases (median age 7.3 years) were older than controls (5.6 years) (p<0.005). The proportion of male patients was higher among cases (57%) than among controls (50%) (p = 0.031; Table 3).

**Table 3:** Characteristics of influenza cases and test-negative controls, Virological Surveillance, Saxony-Anhalt, Germany 2012/13.

	Cases			Controls			
Characteristics	n	exp.	%	n	exp.	%	p-value*
**Male**	347	198	57	487	241	50	0.031
**Vaccinated**	347	33	9.5	487	63	13	0.126
**LAIV** *	329	3	0.91	469	24	5.1	0.001
**TIV** *	329	12	3.7	469	21	4.5	0.562

*chi-squared test, LAIV (live attenuated influenza vaccine), TIV (trivalent inactivated influenza vaccine)

The proportion of patients who were vaccinated did not differ between cases (9.5%) and controls (13%) (p = 0.126). Information about the vaccine type, allowing stratification in LAIV and TIV, was available from 60 (62.5%) of 96 patients. Among these 60 patients, 33 (55%) were vaccinated with TIV and 27 (45%) with LAIV. Influvac (n = 18; Abbott Biol., NL), Afluria (n = 14; CSL Ltd., AU) and Begripal (n = 1; Novartis Vac., I) were used as TIV. There were 15 TIV-vaccinated cases (7x influenza B, 4x A(H3N2), 4x A(H1N1)pdm09) and 3 LAIV-vaccinated cases (1x A(H3N2), 2x A(H1N1)pdm09). The proportion of LAIV-vaccinated patients was smaller among cases (0.91%) than among controls (5.1%, p = 0.001). The proportion of patients vaccinated with TIV did not differ between cases (3.7%) and controls (4.5%) (p = 0.562).

#### Vaccine effectiveness

Adjusted by age group, sex and month of illness, VE of any seasonal vaccine against laboratory-confirmed influenza was 38% (95% CI: 0.8–61%). Among 2–6 year-old and 7–17 year-old children, adjusted VE was 23% (95% CI: -55-62%) and 46% (95% CI: -1.1–72%) respectively (Table 4).

**Table 4:** Vaccine effectiveness of all seasonal vaccines against laboratory-confirmed influenza of all subtypes, stratified by age groups; Multivariable logistic regression, Virological Surveillance, Saxony-Anhalt, Germany 2012/13 (n = 834).

Age group (years)	n	VE (%)	95% CI	p-value
**2–6**	454	23^a^	-55-62	0.465
**7–17**	380	46^a^	-1.1–72	0.054
**total**	834	38^b^	0.8–61	0.046

^a^. VE (vaccine effectiveness) adjusted for month of illness, sex;

^b^. VE adjusted for month of illness, sex, age group

Among children aged 2 to 17 years, the adjusted VE of LAIV was 84% (95% CI: 45–95%), among 2–6 year-old children 90% (95% CI: 20–99%) and among 7–17 year-old children 74% (95% CI: -32-95%). TIV were not significantly effective (Table 5).

**Table 5:** Vaccine effectiveness of live (LAIV) and inactivated vaccines (TIV) against laboratory-confirmed influenza of all subtypes, stratified by age groups; Multivariable logistic regression, Virological Surveillance, Saxony-Anhalt, Germany 2012/13 (n = 798).

		LAIV			TIV		
Age group (years)	n	VE (%)	95% CI	p-value	VE (%)	95% CI	p-value
**2–6**	443	90^a^	20–99	0.030	21^a^	-147-75	0.682
**7–17**	355	74^a^	-32-95	0.106	45^a^	-51-80	0.245
**total**	798	84^b^	45–95	0.004	37^b^	-35-70	0.237

^a^. VE (vaccine effectiveness) adjusted for month of illness, sex;

^b^. VE adjusted for month of illness, sex, age group

Stratification by influenza virus subtypes did not show significant VE for any LAIV or TIV vaccine (Table 6). A trend towards a higher adjusted VE of LAIV against A(H3N2) (84%, 95% CI: -27-98%) and influenza B (no vaccinated cases) was seen among children aged 2–17 years. Adjusted VE of LAIV against A(H1N1)pdm09 was 39% (95% CI: -176-87%). Adjusted VE of TIV was -25% (95% CI: -296-60%) against A(H1N1)pdm09, 63% (95% CI: -67-92%) against A(H3N2) and 39% (95% CI: -66-78%) against influenza B.

**Table 6:** Vaccine effectiveness of live (LAIV) and inactivated vaccines (TIV) against laboratory-confirmed influenza A(H3N2), A(H1N1)pdm09 and B; Multivariable logistic regression, Virological Surveillance, Saxony-Anhalt, Germany 2012/13, age group 2–17 years, (n = 798).

	LAIV			TIV		
Influenza virus subtype	VE (%)	95% CI	p-value	VE (%)	95% CI	p-value
**A/H3N2**	84^a^	-27-98	0.084	63^a^	-67-92	0.197
**A/H1N1pdm09**	39^a^	-176-87	0.518	-25^a^	-296-60	0.699
**B**	-	-	-	39^a^	-66-78	0.333

^a^. VE (vaccine effectiveness) adjusted for month of illness, sex, age group

## Discussion

Our surveillance data measured influenza virus circulation among children and adolescents in Saxony-Anhalt. Compared with our monitoring in previous seasons, the present data indicate a long duration and strong influenza activity of the influenza wave in 2012/13. Among children, influenza B predominated, while A(H3N2) and A(H1N1)pdm09 co-circulated. All characterized influenza viruses were well-matched to the trivalent influenza vaccine strains [27,28]. The detected non-influenza viruses causing ARI in children were RSV and, to a lower extent, adenoviruses, picornaviruses and HMPV.

Our estimates suggest a moderate effectiveness of overall seasonal vaccines against laboratory-confirmed influenza in children. Similar VE estimates against the circulating influenza virus subtypes in 2012/13 were also described by other studies in Europe [29,30]. Distinguishing between LAIV and TIV, our results indicate a high effectiveness of LAIV especially among young children. This high effectiveness could not be achieved by the inactivated vaccines in our study. In fact, TIV tended to have a low effectiveness in preventing influenza among 2–6 year-old children and a moderate VE among 7–17 year-old children. Our results are in line with other studies determining a higher effectiveness of LAIV compared to TIV in children up to 6–7 years of age [2,5–8]. For example, LAIV had a higher effect than TIV in preventing influenza caused by antigenically-matching viral strains in a phase III trial in children aged 6–59 months. Furthermore, open-label studies could show that LAIV was more effective at decreasing the incidence of culture-confirmed influenza illness in young children with recurrent respiratory tract illnesses and in children and adolescents with asthma. Recently, the STIKO recommendations in Germany were changed due to the mentioned and other studies [11]. The preferential use of LAIV is recommended for 2–6 year-old children, but not for older children. Our estimates in 7–17 year-old children indicate a tendency to a better preventive effect of LAIV compared to TIV also in this age group. But we could only determine a non-significant VE of LAIV in older children; this could be due to the smaller sample size. Similar results showing non-significant VE of LAIV in older children were also found in other studies [5, 31,32]. However, it has been also shown that LAIV had a significantly greater relative efficacy compared with TIV in 2–17 as well as 6–17 year-old children [4,32]. LAIV was well tolerated by 6–17 year-old children with asthma [32]. Altogether, literature indicates that LAIV is more effective than TIV among 2–17 year-old children (even with mild to moderate asthma), but this advantage is not seen in adults [2–4,32,33].

A recent simulation study suggested that vaccinating children 2–17 years of age with LAIV is likely associated with a significant reduction in the burden of paediatric influenza in Germany [34]. The authors conclude that annual routine childhood vaccination against seasonal influenza is expected to decrease the incidence of influenza among adults and older people due to indirect effects of herd protection.

Our study is limited by the small number of vaccinated cases, in particular of LAIV-vaccinated cases. When stratifying by influenza virus subtypes, the statistical power was insufficient to produce precise results. Furthermore, information on the date of vaccination was often missing, so that time since vaccination could not be included in our calculations. We could not take into account the number of vaccine doses or previous vaccinations. This may contribute to the low VE of TIV in our study. Studies involving seasonal inactivated influenza vaccines among young children have demonstrated that 2 vaccine doses provide better protection than 1 dose during the first season a child is vaccinated [35]. Further studies found that VE is lower among children aged <5 years who have never received influenza vaccine previously or who received only 1 dose in their first year of vaccination [36–38]. Similar to TIV, a second dose of LAIV is recommended for immunization of vaccine-naive children. However, a clinical trial showed that the efficacy of LAIV after 1 dose was only slightly lower in comparison to 2 doses [39]. A query to chronic underlying diseases was integrated in February 2013 and could not be included in the present study.

Our study was based on a standardized protocol; only data from our surveillance system were included in the analysis. A further strength is that the study’s outcomes were based on sensitive PCR protocols, rather than on culture or respiratory symptoms.

## Conclusions

In summary, our observational study during the first season LAIV was available in Germany suggests that, compared to TIV, LAIV was more effective in preventing laboratory-confirmed influenza, especially among young children. Based on these results, we recommend the use of LAIV for healthy children, for whom LAIV is approved. In addition, we provide evidence for the preferential use of LAIV in 2–6 year-old children as recommended by the STIKO. Further studies are necessary to evaluate our results in coming seasons. We need more data to clarify, if the recommendation should be expanded to include 7–17 year-old children as well.

A related article has been published previously:

Helmeke, Carina: Effektivität der Influenza-Impfung bei Kindern und Jugendlichen in der Saison 2012/2013. Ärzteblatt Sachsen-Anhalt 25 (2014) Ausgabe 1/2, S. 78–80 http://www.aerzteblatt-sachsen-anhalt.de/ausgabe/fachartikel/134-fachartikel-0102-2014/482-effektivitaet-der-influenza-impfung-bei-kindern-und-jugendlichen.html
